# Anticancer Activity of Triterpene Glycosides Cucumarioside A_0_-1 and Djakonovioside A Against MDA-MB-231 as A_2B_ Adenosine Receptor Antagonists

**DOI:** 10.3390/ijms262110327

**Published:** 2025-10-23

**Authors:** Elena A. Zelepuga, Ekaterina A. Chingizova, Ekaterina S. Menchinskaya, Evgeny A. Pislyagin, Sergey A. Avilov, Vladimir I. Kalinin, Dmitry L. Aminin, Alexandra S. Silchenko

**Affiliations:** 1 G.B. Elyakov Pacific Institute of Bioorganic Chemistry, Far Eastern Branch of the Russian Academy of Sciences, Pr. 100-Letya Vladivostoka 159, 690022 Vladivostok, Russia; zel@piboc.dvo.ru (E.A.Z.); ekaterinamenchinskaya@gmail.com (E.S.M.); pislyagin@hotmail.com (E.A.P.); kalininv@piboc.dvo.ru (V.I.K.); daminin@piboc.dvo.ru (D.L.A.); 2 Department of Biomedical Science and Environmental Biology, Kaohsiung Medical University, Kaohsiung 80708, Taiwan

**Keywords:** adenosine receptors, A_2B_AR, triterpene glycosides, MDA-MB-231, triple-negative breast cancer (TNBC), anticancer, antagonist, MAPK, molecular docking and dynamics

## Abstract

Breast cancer is the most prevalent cancer in women worldwide and presents a major therapeutic challenge, particularly triple-negative breast cancer (TNBC), a subtype characterized by an aggressive clinical course but heightened sensitivity to chemotherapy. Natural products, such as triterpene glycosides derived from sea cucumbers, have emerged as promising candidates with high anticancer potential against TNBC. This study investigated the pathways of anticancer action of cucumarioside A_0_-1 (Cuc A_0_-1) and djakonovioside A (Dj A), isolated from the sea cucumber *Cucumaria djakonovi*, triggered and regulated in MDA-MB-231 cells (triple-negative breast cancer cell line). We employed functional assays (cAMP level, Ca^2+^ influx, control of cell proliferation and colony formation), Western blotting for mitogen-activated protein kinase MAPK) signaling, and in silico molecular docking. A_2B_ adenosine receptor (A_2B_AR) was identified as a novel target for both glycosides. As antagonists, they reduced cAMP production and inhibited NECA (5-(N-ethylcarboxamido)adenosine)-induced Ca^2+^ influx. This A_2B_AR blockade suppressed the MAPK pathway, profoundly inhibiting phospho-ERK1/2, p38, and JNK1/2, which led to the activation of the intrinsic apoptotic pathway and strong inhibition of cell proliferation and colony formation. Surprisingly, co-treatment with the NECA agonist enhanced the antiproliferative effects of the glycosides. It was supposed that the interaction of glycosides with the NECA-preactivated receptor may bias signaling toward the Gi and Gq/PLC/ERK1/2 pathways, underscoring the central role of the MAPK pathway in controlling cell growth. Molecular docking confirmed binding to the A_2B_AR orthosteric site, revealing that Cuc A_0_-1 and Dj A employ distinct interaction modes. To our knowledge, this is the first report to define A_2B_AR as a target for sea cucumber glycosides. Their potent antitumor effects, mediated through the antagonism of A_2B_AR and subsequent MAPK pathway inhibition, position them as promising lead compounds for cancer types with high expression A_2B_AR.

## 1. Introduction

Adenosine receptors (ARs) are a family of transmembrane receptors coupled with G proteins that bind to the endogenous ligand, adenosine. Currently, four adenosine receptor subtypes have been distinguished: A1, A_2A_, A_2B_, and A3 [1,2]. These receptors are essential components of the body’s regulatory system and influence a wide range of physiological and pathological processes. Adenosine receptors have recently emerged as promising new targets for antitumor therapy, with numerous studies highlighting their potential for novel anticancer drug development [3,4,5,6,7]. Although the A_2B_ adenosine receptor (A_2B_AR) is constitutively expressed at low density across most cell types, its expression is highly induced under pathological conditions such as hypoxia, inflammation, and cancer. This overexpression is particularly prominent in immune cells (macrophages, dendritic cells, T cells, and B cells) and numerous cancer cell types [8]. High A_2B_AR expression has been found in many human tumor cell lines, including MDA-MB-231 breast cancer cells [9,10].

Adenosine receptors differ in the type of G proteins they recruit and in the downstream signaling pathways activated. A_2B_ARs are primarily linked to Gs proteins and therefore stimulate Adenylyl Cyclase (AC) and protein kinase A (PKA) activity. However, they also interact with the Gq family of G-proteins to activate phospholipase C (PLC) and protein kinase C (PKC) and increase intracellular calcium levels [11,12]. Moreover, the G protein coupling of the receptor is cell type-dependent [12]. The A_2B_AR has recently been reported to trigger intracellular calcium mobilization in the MDA-MB-231 cell line through Gi, Gs, and Gq proteins [13].

A_2B_ARs coupled to multiple signaling pathways mediate both beneficial and undesirable effects. Despite the well-known pro-tumoral role of A_2B_ARs in cancer cells, they can also exert an inhibitory effect on tumor cell proliferation [14] mediated through the reduction in ERK1/2 phosphorylation (i.e., via the MAPK pathway). It has been shown that cAMP and Ca^2+^ signals are sufficient for the reduction in ERK1/2 phosphorylation in MDA-MB-231 cells [7]. As the receptor plays an important role in tumor cell proliferation, angiogenesis, metastasis, and immune suppression, A_2B_AR antagonists are potential therapeutic agents for various types of cancers. A_2B_AR antagonist was shown to reduce tumor burden via immune mechanisms, while activation of this receptor increased metastasis in mouse models [15,16,17,18]. Thus, the development of new modulators of A_2B_AR to regulate pathological reactions in the tumor microenvironment is of great clinical significance for the treatment of cancer-related complications, including TNBC.

**Figure 1:**
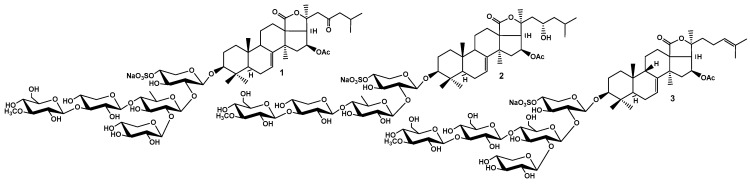
Structures of triterpene glycosides from the sea cucumber *Cucumaria djakonovi*: **1**—cucumarioside A_0_-1; **2**—djakonovioside A; **3**—djakonovioside D_1_.

Recently, we showed that micromolar concentrations of Cuc A_0_-1 and Dj A—triterpene glycosides from *Cucumaria djakonovi* (Figure 1)—suppress the proliferation and migration, reduce or completely block colony formation of MDA-MB-231 cancer cells [19]. Moreover, these compounds induce ROS formation and cause depolarization of mitochondrial membranes, leading to cell cycle arrest and changes in the expression levels of proteins regulating the cell cycle and apoptosis in TNBC cells [20]. These events led to the initiation of the mitochondrial caspase-dependent apoptosis pathway. Notably, the MAPK signaling pathway is upstream of mitochondrial-mediated apoptosis. Therefore, it was proposed that these downstream processes are initiated through the interaction of the tested compounds with the membrane target A_2B_AR, which is highly expressed on the membranes of MDA-MB-231 cells. To test this hypothesis, we determined whether the glycosides Cuc A_0_-1 and Dj A modulate the ERK1/2, JNK1/2, and p38 MAPK signaling pathways downstream of adenosine receptor activation. First, we characterized the effects of glycosides and the standard A_2B_AR non-selective agonist, NECA (N-ethylcarboxamidoadenosine), and the selective A_2B_AR antagonist, PSB-1115 (1-Propyl-8-(4-sulfophenyl)xanthine potassium salt hydrate), on cAMP and Ca^2+^ levels in MDA-MB-231 cells.

As computational chemistry is now an integral part of drug discovery, design, and development, structural drug design was applied. This method uses the 3D structure of the target protein (receptor) to identify potential ligands that fit into the active site of the protein. Molecular docking and dynamics were implemented to model the binding of glycosides with the membrane target, the human A_2B_ adenosine receptor.

## 2. Results

### 2.1. The Influence of the Glycosides on cAMP Levels

**Figure 2:**
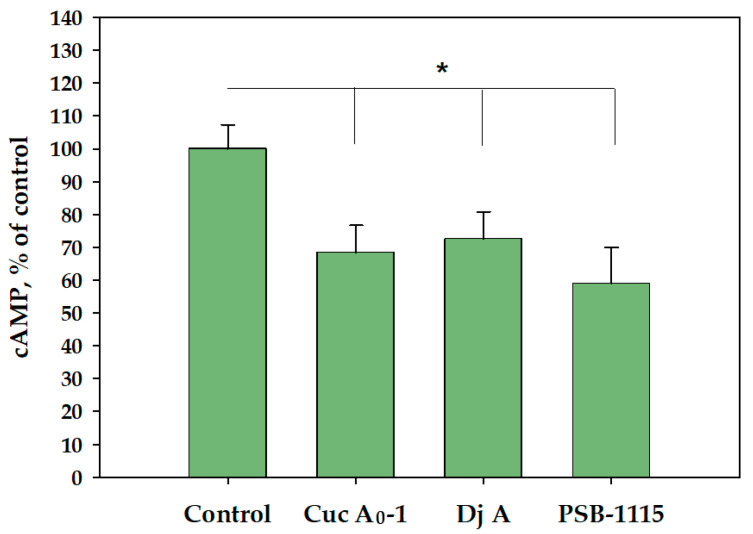
Evaluation of cAMP content in MDA-MB-231 cells after treatment with Cuc A_0_-1 (1 μM), Dj A (2 μM), and PSB-1115 (1 μM) for 6 h using an ELISA kit. * *p* < 0.05 compared with control (untreated MDA-MB-231 cells).

The initial stage of our research was to study the effect of glycosides on the level of cAMP, the first secondary messenger produced in TNBC cells as a result of A_2B_AR modulation. When MDA-MB-231 cells were treated with the glycosides Cuc A_0_-1 (1 µM) and Dj A (2 µM) and the selective A_2B_AR antagonist PSB-1115 (1 µM) for 6 h, we observed a decrease in cAMP levels of 31, 27, and 41%, respectively, compared to untreated cells (Figure 2).

### 2.2. Effect of Glycosides on Intracellular Calcium Levels in MDA-MB-231 Cells

**Figure 3:**
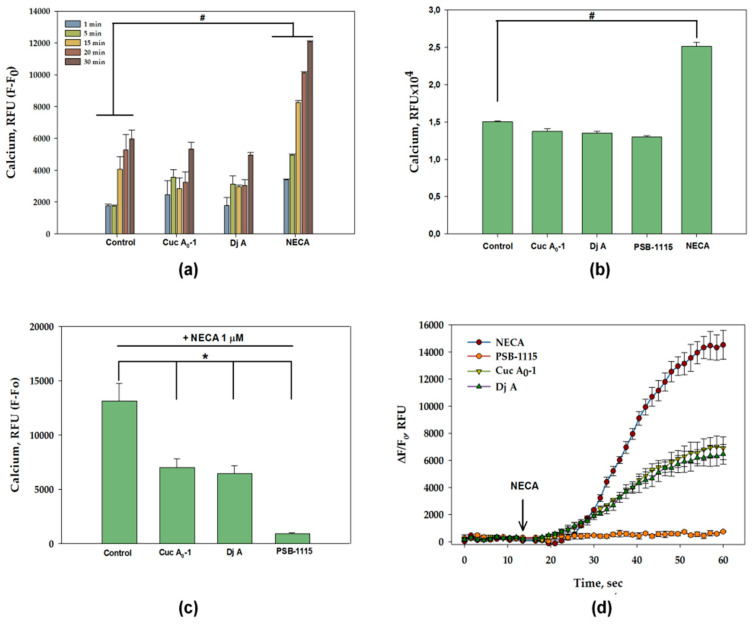
The influence of compounds on intracellular calcium levels in MDA-MB-231 cells: effect of Cuc A_0_-1 (1 µM), Dj A (2 µM) and NECA (1 µM) on Ca^2+^ influx in time (**a**), and at 30 min (**b**); effect of Cuc A_0_-1 (1 µM), Dj A (2 µM), PSB-1115 (1 µM) on Ca^2+^ influx induced by NECA (1 µM) (**c**); representative curves of [Ca^2+^]i elevation induced by NECA (1 µM) alone or in the presence of Cuc A_0_-1 (1 µM), Dj A (2 µM) or PSB-1115 (1 µM) (**d**). Data are presented as m ± SE (n = 6). * *p* < 0.05 compared to NECA, # *p* < 0.05 compared to control.

As activation of A_2B_AR causes an increase in intracellular calcium [9,13], the ability of glycosides, NECA, and PSB-1115 to induce intracellular exposure of Ca^2+^ in the TNBC cell line MDA-MB-231 was investigated using the calcium-selective fluorescent probe FluoriCa-8 AM. Cuc A_0_-1 (1 µM), Dj A (2 µM), and PSB-1115 (1 µM) did not cause intracellular Ca^2+^ concentration elevation, whereas NECA (1 μM) led to a significant increase in intracellular calcium levels in TNBC cells (Figure 3a,b). Moreover, NECA could not stimulate the receptor and increase Ca^2+^ levels inside cells after the pretreatment of MDA-MB-231 cells with the studied glycosides for 30 min, because the subsequent addition of the agonist (NECA, 1 μM) was unable to fully restore calcium influx (Figure 3c,d). Thus, Cuc A_0_-1 (1 µM) reduced the influx of calcium induced by the agonist NECA by 52% and Dj A (2 µM) by 48%, demonstrating comparable A_2B_AR inhibition. Calcium influx was also effectively suppressed by the standard blocker PSB-1115 (1 μM) (Figure 3c,d).

### 2.3. Influence of Glycosides on p38/p-p38, ERK1/2/p-ERK1/2, JNK1/2/p-JNK1/2 in MDA-MB-231 Cells

The effects of Cuc A_0_-1, Dj A, NECA, and PSB-1115 on the expression of serine/threonine kinases in MDA-MB-231 cells were evaluated using Western Blotting. Specifically, we analyzed the mitogen-activated protein kinases (MAPKs) p38, ERK1/2, and JNK1/2, which play a key role in transducing extracellular signals that regulate various cellular responses, including proliferation, differentiation, and apoptosis.

**Figure 4:**
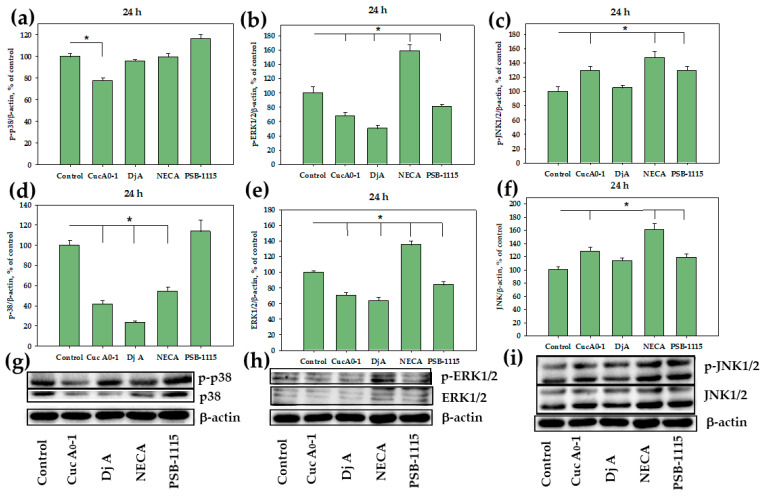

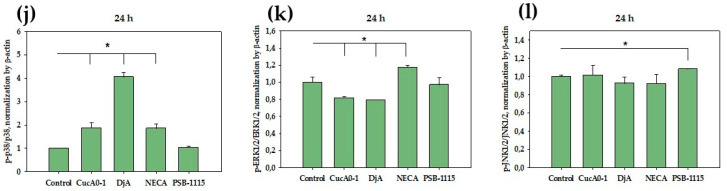
Effect of Cuc A_0_-1 (1 µM), Dj A (2 µM), PSB-1115 (1 µM) and NECA (1 µM) on p38/p-p38, ERK1/2/p-ERK1/2, JNK1/2/p-JNK1/2 in MDA-MB-231 cells treated for 24 h. Normalization of p-p38 (**a**), p-ERK1/2 (**b**), p-JNK1/2 (**c**), p-38 (**d**), ERK1/2 (**e**), and JNK (**f**) levels to the housekeeping protein β-actin. Western Blot results of p38 and p-p38 (**g**), ERK1/2 and p-ERK1/2 (**h**), JNK1/2 and p-JNK1/2 (**i**) levels. Effect of Cuc A_0_-1 (1 µM), Dj A (2 µM), PSB-1115 (1 µM) and NECA (1 µM) on protein expression ratio p-p38/p38 (**j**), p-ERK1/2/ERK1/2 (**k**), and p-JNK1/2/JNK1/2 (**l**) in MDA-MB-231 cells treated for 24 h, normalized to β-actin. Protein levels were normalized to those of the control group (untreated cells). * *p* < 0.05 compared to control (untreated MDA-MB-231 cells).

24 h exposure of MDA-MB-231 cells to 1 µM Cuc A_0_-1 reduced total p38 and phospho-p38 (p-p38) levels by 60% and 22%, respectively. In this case, the expression ratio of p-p38/p38 increased twofold compared to that in the control, as in the case of NECA (Figure 4j). Dj A (2 µM) elicited a more pronounced decrease in total p38 by 76%, without having a significant effect on the level of p-p38. Therefore, we observed a fourfold increase in the p-p38/p38 expression ratio compared to that of the control. Treatment with NECA (1 µM) for 24 h also resulted in a decrease in total p38 by 46%, without affecting the level of p-p38. When the receptor was blocked by PSB-1115, the decrease in the expression of p-38 and its phosphorylated form was not observed (Figure 4a,d,g), the expression ratio of p-p38/p38 was comparable to that of the control (Figure 4j). Additionally, 24 h of treatment of TNBC cells with Cuc A_0_-1 and Dj A reduced the levels of total ERK1/2 by 30% and 37% and the phosphorylated form of ERK1/2 by 33% and 50%, respectively. Treatment with PSB-1115 for the same duration also led to a decrease in total and phosphorylated ERK1/2 by 17% and 19%, respectively. NECA treatment of MDA-MB-231 cells led to a 35% and 58% increase in the total and phosphorylated forms of ERK1/2, respectively (Figure 4b,e,h). Treatment of MDA-MB-231 cells with glycosides for 24 h resulted in a 20% decrease in the p-ERK1/2/ERK1/2 expression ratio. NECA increased the p-ERK1/2/ERK1/2 expression ratio by 18%. PSB-1115 had no effect on the ratio p-ERK1/2/ERK1/2 (Figure 4k). Only a slight increase (28%) in total and activated JNK1/2 was caused by Cuc A_0_-1 in MDA-MB-231 cells, while Dj A did not cause significant changes in the JNK1/2 level in the cells. NECA treatment resulted in the upregulation of both phosphorylated (47%) and total (61%) JNK1/2. At the same time, PSB-1115 led to an insignificant increase in total JNK1/2 and a slight increase in the phosphorylated forms of JNK1/2 by 28% (Figure 4c,f,i). When MDA-MB-231 cells were treated for 24 h with glycosides and NECA, the level of the expression ratio of p-JNK1/2/JNK1/2 did not change, whereas under the action of the antagonist PSB-1115, the level of expression of p-JNK1/2/JNK1/2 increased (Figure 4l).

**Figure 5:**
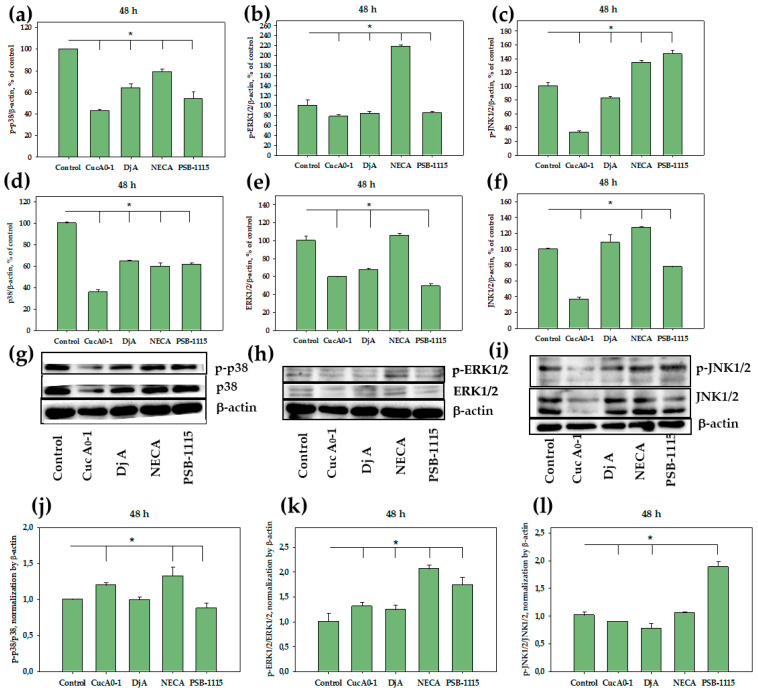
Effect of Cuc A_0_-1 (1 µM), Dj A (2 µM), PSB-1115 (1 µM) and NECA (1 µM) on p38/p-p38, ERK1/2/p-ERK1/2, JNK1/2/p-JNK1/2 in MDA-MB-231 cells treated for 48 h. Normalization of p-p38 (**a**), p-ERK1/2 (**b**), p-JNK1/2 (**c**), and p38 (**d**), ERK1/2 (**e**), JNK1/2 (**f**) levels relative to the housekeeping protein β-actin. Western Blot results of p38 and p-p38 (**g**), ERK1/2 and p-ERK1/2 (**h**), JNK and p-JNK (**i**) levels. Effect of Cuc A_0_-1 (1 µM), Dj A (2 µM), PSB-1115 (1 µM) and NECA (1 µM) on protein expression ratio p-p38/p38 (**j**), p-ERK1/2/ERK1/2 (**k**), p-JNK1/2/JNK1/2 (**l**) in MDA-MB-231 cells treated for 48 h, normalized to β-actin. Protein levels were normalized to the control group (untreated cells). * *p* < 0.05 compared to control (untreated MDA-MB-231 cells).

Furthermore, we assessed the effects of these compounds at the same concentrations on MAPKs in TNBC cells over a prolonged 48-hour incubation period. Cuc A_0_-1 inhibited p38 by 65% and p-p38 by 58%, whereas Dj A reduced the levels of p38 by 35% and p-p38 by 36%. NECA decreased total p38 by 40% and p-p38 by 21%, while PSB-1115 reduced the level of total p38 by 39% and its activated form by 46% (Figure 5a,d,g). When MDA-MB-231 cells were treated with Cuc A_0_-1 for 48 h, the p-p38/p38 expression ratio increased. Dj A did not cause a significant change in the expression ratio, whereas the antagonist PSB-1115 significantly decreased the expression level of p-p38/p38 (Figure 5j). Triterpene glycosides reduced the levels of both total ERK1/2 and p-ERK1/2: the effect of Cuc A_0_-1 was 41% in relation to ERK1/2 and 22% in relation to p-ERK1/2, and the effect of Dj A was 32% toward ERK1/2 and 26% toward p-ERK1/2 compared to untreated cells. PSB-1115 affected the ERK1/2 level in a similar manner to the glycosides, to a greater extent reducing the level of total ERK by 50% and p-ERK1/2 by 15%. Notably, NECA strongly increased p-ERK1/2 by 118% over 48 h, while having no significant effect on the level of total ERK1/2 (Figure 5b,e,h). Exposure of MDA-MB-231 cells to glycosides and PSB-1115 for 48 h resulted in an increase in the p-ERK1/2/ERK1/2 expression ratio, with overall inhibition of both p-ERK1/2 and ERK1/2. Exposure to NECA resulted in a twofold increase in the p-ERK1/2/ERK1/2 expression ratio without inhibiting these proteins (Figure 5k). Cuc A_0_-1 significantly decreased the levels of total and activated JNK1/2 by 64% and 67%, respectively. Dj A slightly inhibited p-JNK1/2 by 18% and insignificantly increased total JNK1/2 levels. NECA increased the levels of both activated and total JNK1/2 by 34% and 27%, respectively. PSB-1115 led to the accumulation of p-JNK (47%), while decreasing the level of total JNK by 22% (Figure 5c,f,i). Exposure of MDA-MB-231 cells to glycosides for 48 h resulted in a significant decrease in the p-JNK1/2/JNK1/2 expression ratio, with an overall inhibition of JNK1/2 expression. PSB-1115 treatment resulted in a two-fold increase in the p-ERK1/2/ERK1/2 expression ratio. NECA had no effect on the expression ratio of these proteins (Figure 5l).

### 2.4. Effect of Glycosides on MDA-MB-231 Cell Proliferation

**Figure 6:**
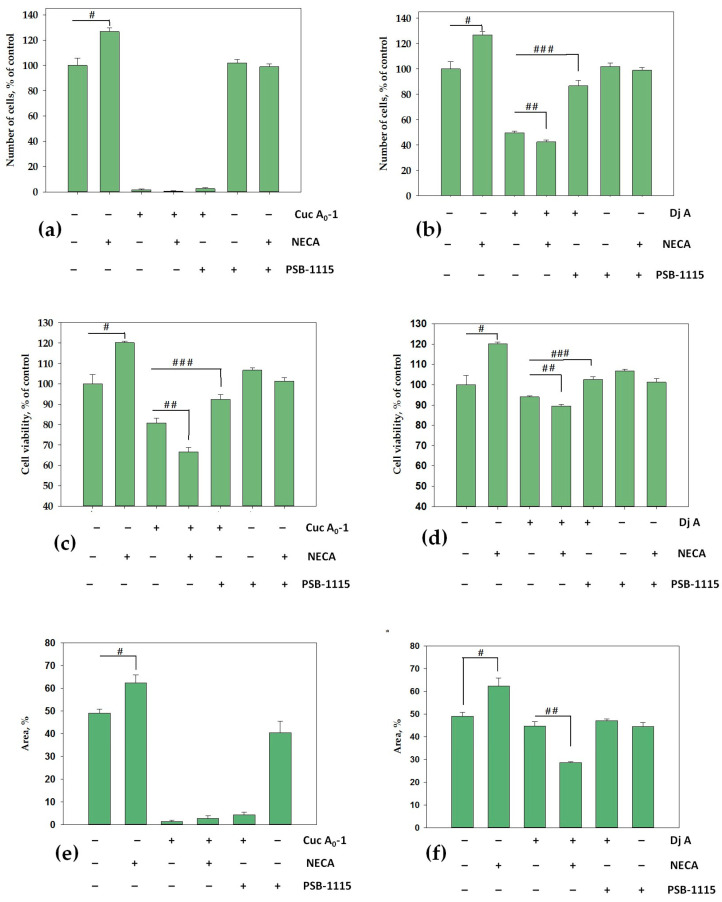
Effect of Cuc A_0_-1 (1 μM) (**a**) and Dj A (2 μM) (**b**) on MDA-MB-231 cell proliferation for 9 days using the cell counting method; effect of Cuc A_0_-1 (1 μM) (**c**) and Dj A (2 μM) (**d**) using the MTS method. All figures show the effects of the standard AR agonist NECA (1 μM) and A_2B_AR antagonist PSB-1115 (1 μM), as well as their combined effects with glycosides. The influence of Cuc A_0_-1 (1 μM) (**e**) and Dj A (2 μM) (**f**), with or without NECA (1 μM) and PSB-1115 (1 μM), on the formation and growth of MDA-MB-231 cell colonies for 14 days. # *p* < 0.05 compared to control untreated MDA-MB-231 cells; ## *p* < 0.05 compared to the difference between glycosides and glycosides + NECA; ### *p* < 0.05 compared to the difference between glycosides and glycosides + PSB-1115.

It is known that treatment with adenosine receptor agonists promotes the proliferation of MDA-MB-231 cells [21]. We studied the influence of the AR agonist NECA, A_2B_AR antagonist PSB-1115, and the tested compounds on the proliferation of MDA-MB-231 cells using different approaches. First, the number of cells was counted after 9 days of exposure. NECA enhanced cell proliferation by 27%, whereas Cuc A_0_-1 inhibited it by 98%. As a result of the combined action of Cuc A_0_-1 and NECA, an antiproliferative effect was observed, with almost complete blockage of cell growth (99%). The combined action of Cuc A_0_-1 and PSB-1115 also demonstrated a significant antiproliferative effect of 97% (Figure 6a). PSB-1115 alone did not affect cell proliferation, consistent with the literature data [22]. Moreover, the effect of NECA was neutralized by the antagonist PSB-1115, and the resulting proliferation was comparable to that of untreated cells (Figure 6a,b). Dj A was less active and inhibited proliferation by 50%; in combination with NECA, the antiproliferative effect of the glycoside increased to 58%. However, the joint action of Dj-A and PSB-1115 resulted in a synergistic effect that strengthened the proliferation of MDA-MB-231 cells to 86% from 50% under the action of Dj A alone (Figure 6b).

Second, the influence of the compounds on proliferation was tested using the MTS method after 9 days of exposure. The results demonstrated that NECA led to an increase in proliferation by 20%, whereas Cuc A_0_-1 and Dj A inhibited proliferation by 20% and 6%, respectively (Figure 6c,d). The combined action of Cuc A_0_-1 and NECA increased the antiproliferative effect to 34%, whereas the combined action of Cuc A_0_-1 and PSB-1115 significantly decreased the antiproliferative effect to 8%, which was comparable to that of the control cells (Figure 6c). The combined action of Dj A and NECA led to an increase in the antiproliferative activity from 6% to 11%, whereas the combined action of Dj A with PSB-1115 restored the proliferation of MDA-MB-231 cells to control values (Figure 6d). The results of the MTS method in relation to the action of PSB-1115 alone and in combination with NECA were the same as those obtained using the cell counting method (Figure 6a–d).

Third, the effect of the compounds on colony formation and growth of TNBC cells (MDA-MB-231) was investigated after 14 days of incubation. In the control (untreated cells), the overgrowth of the well area was 49%. In the presence of NECA (1 μM), the area occupied by cell colonies increased by 27% compared to that in the control (Figure 6e,f). Incubation of cells with PSB-1115 (1 μM) reduced the number of colonies by 16% compared to the control. Treatment of MDA-MB-231 cells with Cuc A_0_-1 (1 μM) resulted in a pronounced inhibition of overgrowth by 97% (Figure 6e). Dj A was much less active and reduced the area occupied by cell colonies by 10% (Figure 6f) compared to the control. When PSB-1115 and Cuc A_0_-1 were added together, they strongly inhibited colony growth, and the overgrowth area was only 8%. This effect was almost not observed when Dj A and PSB-1115 were used together. Simultaneous addition of NECA and Dj A led to significant inhibition of colony growth, up to 42% compared to the control (Figure 6f). The area of cell colonies treated by NECA together with Cuc A_0_-1 was only 6% due to the strong antiproliferative action of the glycoside (Figure 6e).

Therefore, we demonstrated using several independent methods that the adenosine receptor agonist NECA enhances MDA-MB-231 cell proliferation, and that the combination of NECA or the selective A_2B_AR antagonist PSB-1115 with the glycosides inhibited cell growth and division, but to different extents. Thus, upon NECA-induced activation of adenosine receptors, an increase in the antiproliferative effect of glycosides was observed. The addition of an antagonist to the cells weakened the antiproliferative effect of glycosides alone.

### 2.5. In Silico Modeling of the Glycosides Binding with A_2B_ Adenosine Receptor (A_2B_AR)

A_2B_AR is a member of the G-protein-coupled receptor superfamily, with seven transmembrane α-helical structures (TMs), an extracellular amino terminus, and an intracellular carboxyl terminus coupled to G-proteins. The N-terminal domain has N-glycosylation sites that influence the trafficking of the receptor to the plasma membrane [23]. Literature data on the mode of action of the A_2B_AR predominantly derived from molecular modeling but not based on the structure established experimentally [24,25,26]. Currently, only four structures of A_2B_AR ligand-bound states, determined by cryogenic electron microscopy (cryo-EM), are available, resolved at 2.8 or 3.2 Å. Moreover, data on the extracellular loops (ECL2 and ECL3) of these structures are lacking.

These loops may affect the interaction of the glycosides with the receptor, and the full-length A_2B_AR model, including the missing extracellular loops and the intracellular loop (ICL3), was constructed based on the structure of this receptor coupled to Gs-protein (PDB ID 8HDP) using the Loop Modeling Tool of the MOE 2019.01 CCG package [27]. Next, the A_2B_AR model was embedded into an asymmetric mammalian plasma membrane using the insertion method on CHARMM-GUI web server. [28,29]. To predict the most likely mechanisms of Cuc A_0_-1, Dj A, and djakonovioside D_1_ (Dj D_1_) [30] (used as a negative control because it is not cytotoxic against cancer cells) interactions with the adenosine receptor, molecular docking was performed using the MOE 2019.01 CCG package [27]. The most energetically favorable glycoside positions were selected from 30,000 solutions for further investigation. To characterize the binding site of the receptor for each glycoside, docking pose enhancement with 20 ns short-term all-atomic MD simulations was performed, allowing the consideration of protein flexibility.

Cuc A_0_-1 and Dj A shared a binding site with known agonists and antagonists of A_2B_AR—orthosteric binding site [31] and were estimated to have comparable binding energies of −33.77 kcal/mol and −28.64 kcal/mol, respectively. The evaluation of the conformational flexibility of the components of the complexes showed only slight changes in the protein backbone position during the simulation, with RMSD_bb_ values ranging from 1.13 to 1.75 Å for all A_2B_AR residues, indicating the validity of the generated binding sites for glycosides. Moreover, the interface surface areas of Cuc A_0_-1 and Dj A with A_2B_AR in the membrane environment and the complementarity of the ligand and receptor surfaces increased slightly during the short-term MD simulation (Table S1), indicating the stability of the predicted complexes. Cuc A_0_-1 entered more densely and deeply into the orthosteric site of A_2B_AR (Figure S1) and was characterized by a SASA loss of 746.7 Å^2^ and a total shape complementarity (S_compl_) of 0.71 upon complex formation, compared to 605.8 Å^2^ and 0.68, respectively, for Dj A, which was associated primarily with the A_2B_AR allosteric site and surrounding lipids.

However, the modes of binding to the receptor were fundamentally different for Cuc A_0_-1 and Dj A.

**Figure 7:**
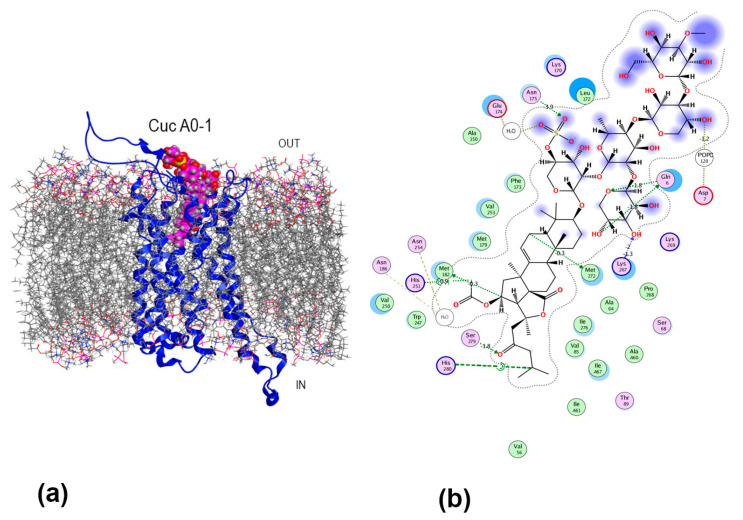
Structural diagram (**a**) of the Cuc A_0_-1 complex with A_2B_AR in a lipid environment and 2D intermolecular interaction scheme (**b**). A_2B_AR is presented as a blue ribbon, asymmetric mammalian plasma membrane lipids—as gray sticks, Cuc A_0_-1 as magenta balls. The aqueous environment and some lipid membrane components were removed for clarity.

Cuc A_0_-1 adopted a specific conformation within the receptor complex, enabling its aglycone to penetrate deeply into the binding site (Figure 7a). This site largely overlaps with adenosine and the known A_2B_AR synthetic agonist BAY 60-6583 (PDB ID 8HDP and 8HDO) sites [32,33]. In the ligand–receptor complex, the aglycone side chain was linked to the receptor’s TM7 via the hydrogen bond (HB) formed by the 23-keto group with Ser279 (−2.1 kcal/mol), which is a crucial residue for the nucleoside’s interactions with the A_2B_AR [25] and π-alkyl interactions, while 16-*O*-acetic group formed the HBs network via both direct bonding with His251 (−0.9 kcal/mol) and mediated bridging HBs through conserved water molecules with Asn254 and Asn186 localized on TM6 and TM5 helixes. Cuc A_0_-1 aglycone also promoted complex formation by HBs generated with the conservative residues Met182 on the TM5 helix, which connects every A_2A_AR ligand for which the structure has been solved, and Met272 on TM7 (Figure 7b). In addition to HBs, the aglycone anchored inside the orthosteric binding site by multiple high-energy Van der Waals (VdW) interactions forming tight contacts with the residues Met272, Val250, Ile276, Ile67, Met179, Phe173, Val85, Val253, Thr87, and Ala64 with the energy contribution ranges from −1.1 kcal/mol up to −3.27 kcal/mol for each those can be considered as “hot spots” for the formation of the complex.

The carbohydrate moiety of Cuc A_0_-1 tightly fastened the glycoside in the area of the allosteric site. The sulfate group at the first xylose residue is attached to ECL2 via a strong HB with Asn175 and Glu174 through a water-mediated bridging HB interaction. The fifth xylose residue attached to TM1 formed two HBs with Glu6 (total contribution of up to −3.6 kcal/mol) and to ECL3 through HBs with Lys267, contributing −3.3 kcal/mol. These residues presumably determine the ARs ligand selectivity. It should be noted that in the complex, the Cuc A_0_-1 aglycone nucleus and the first two monosaccharide residues were quite stable, with an RMSF of less than 1 Å. In contrast, the side chain of the aglycone and the terminal monosaccharide residues demonstrated some flexibility (RMSF values of 2.24 Å and 1.48 Å, respectively) (Figure S2).

**Figure 8:**
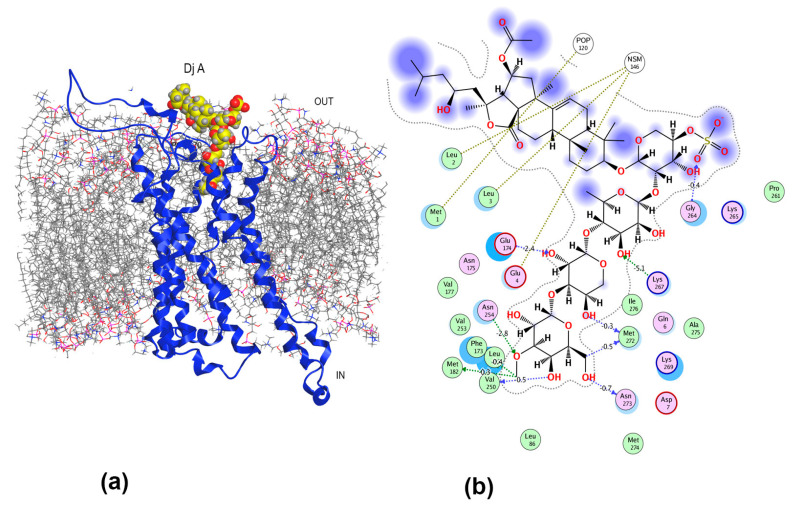
Structural diagram (**a**) of Dj A complex with A_2B_AR in a lipid environment and 2D intermolecular interaction scheme (**b**). A_2B_AR is presented as a blue ribbon, asymmetric mammalian plasma membrane lipids—as gray sticks, Dj A as red and yellow balls. The aqueous environment and some lipid membrane components were removed for clarity.

The results of docking followed by short-term MD simulations revealed that Dj A also bound to the orthosteric site of A_2B_AR, although it demonstrated a different mode of localization (Figure 8a) compared to Cuc A_0_-1. The carbohydrate chain of Dj A was placed inside the binding pocket but did not penetrate deeply, as the glycoside was located to a greater extent in the area of the allosteric site of A_2B_AR. The “hot spots” of the glycoside/receptor interaction were Val250 on TM6 at the orthosteric site and allosteric site residues Leu172 and Lys267 on ECL2 and ECL3, respectively (Figure 8b). The contributions of close VdW contacts of terminal 3-*O*-methylglucose residue of Dj A and Val250 as well as HB between the hydroxyl group at C-4 of this residue and Val250 were −2.06 kcal/mol and −0.70 kcal/mol, respectively. Similarly, Leu86, Phe173, Val253, and Met182 stabilized this sugar residue at the binding pocket through VdW interactions contributing from −1.10 to −1.38 kcal/mol each, as well as the HBs of the 3-*O*-methyl group with Asn254, Met182 and Phe173 (−2.8 kcal/mol, −0.30 kcal/mol and −0.40 kcal/mol, respectively). In addition, the CH_2_OH-group of the terminal sugar residue formed HBs with Asn273 and Met 272. The third (xylose) residue of Dj A was attached to ECL2 and TM7 through HBs of the hydroxyl groups at C-2 and C-4 with Glu174 (−2.4 kcal/mol) and Met272, respectively. Quinovose residue (the second in the chain) strongly electrostatically interacted (−5.1 kcal/mol) with the allosteric site residue Lys267 and HBs formed between the sulfate group and allosteric site Gly264 residue. This extensive interaction network strongly anchors glycoside within both the orthosteric and allosteric sites. Dj A interacts with functionally important receptor residues, including conserved (Asn254 (TM6), Phe173 (ECL2), and Met182 (TM6)), which are involved in the modulation of several AR subtypes by agonists and antagonists, and variable residues Val250 and Val253 (TM6), Gly264, and Lys267 (ECL3), which are unique to the A_2B_AR subtype and are involved in the selectivity of ligand recognition.

**Figure 9:**
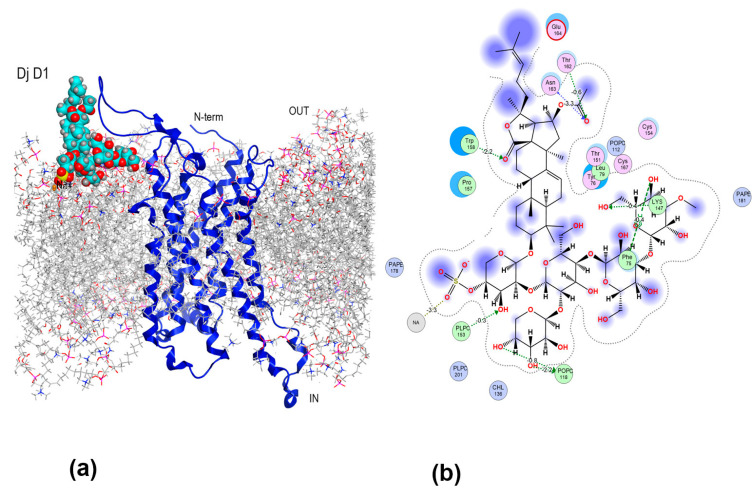
Structural diagram (**a**) of Dj D_1_ binding to A_2B_AR in a lipid environment and 2D intermolecular interaction scheme (**b**). A_2B_AR is represented as a blue ribbon, asymmetric mammalian plasma membrane lipids as gray sticks, and Dj D_1_ as cyan balls. Aqueous environment and some of the lipid membrane components are removed for clarity.

The binding of Dj D_1_ (Figure 1), which was not toxic to MDA-MB-231 cells at the tested concentrations [30], to A_2B_AR was modeled for comparison with the modes of binding of the active glycosides. Dj D_1_ preferentially bound to the lipid membrane and occupied the space between the membrane surface and ECL2 of A_2B_AR (Figure 9a). Although this position was not among the top positions of energetically efficient docking solutions, this structure was chosen for further analysis to determine whether Dj D_1_ modulates A_2B_AR. The glycoside formed non-covalent interactions preferably by its carbohydrate moiety with an estimated binding energy of −15.26 kcal/mol. The sulfate group attached firmly to the PAPE and PLPC lipid heads contributing −3.21 kcal/mol and −2.29 kcal/mol, respectively (Figure 9b). The branched carbohydrate moiety can directly bind to the transmembrane helix 3 (TM3) of A_2B_AR by H-π interaction of the OH-group at C-5 of the 3-*O*-methylglycose unit with the Phe756-ring (−0.5 kcal/mol) as well as by VdW contact of the hydroxymethylene group of the second glucose unit with Tyr76. Additionally, Dj D_1_ interacted with the neighboring lipid POPC through hydrogen bonds (HBs) of OH-groups at C-3 and C-4 of the fifth (xylose) residue (−2.2 kcal/mol and −0.4 kcal/mol), which in turn formed π-anion interactions with Phe80 of A_2B_AR. The 16-*O*Ac and oxo-group of 18(20)-lactone as the H-acceptors formed an extensive network of HBs with Asn163 (−3.3 kcal/mol), Trp158 (−2.2 kcal/mol), Thr162 (−0.6 kcal/mol), and Lys147 (−0.4 kcal/mol) stabilizing the glycoside in the complex. Nevertheless, the glycoside did not penetrate either the orthosteric or allosteric binding sites of A_2B_AR.

## 3. Discussion

A_2A_AR and A_2B_AR antagonists have been shown to reduce tumor metastasis in various mouse models of melanoma, ovarian, bladder, and breast cancers [17,34]. It is well established that G protein-coupled receptors can transduce effects through more than one signaling pathway. A_2B_AR activation initiates both the Gs/adenylyl cyclase (AC)/cAMP and Gq/phospholipase C/Ca^2+^ signaling pathways to mediate its physiological effects. The mode of G protein coupling is not only cell type-dependent but can also cause diverse cell responses, such as anti- or pro-inflammatory responses [12]. A_2B_AR-mediated activation MAPK [35,36,37,38] promotes intrinsic apoptosis by phosphorylating Bcl-2 and Bax [39]—these same proteins are also modulated by the glycosides Cuc A_0_-1 and Dj A [20].

In MDA-MB-231 cells, the glycosides Cuc A_0_-1 and Dj A reduced cAMP levels, exhibiting an effect similar to—though weaker than—the selective A_2B_AR antagonist PSB-1115 [40]. This suggests that they act as A_2B_AR antagonists that inhibit Gs coupling and adenylyl cyclase activation.

We demonstrated that exposure of MDA-MB-231 cells to the AR agonist NECA significantly increased intracellular calcium levels. This effect was halved by the glycosides Cuc A_0_-1 and Dj A and completely abolished by the selective A_2B_AR antagonist, PSB-1115. These data indicate that glycosides also inhibit Gq-coupling of A_2B_AR, further supporting their mode of action as receptor antagonists.

The MDA-MB cell lines, particularly MDA-MB-231 and MDA-MB-468, are used in cancer research to study the role of p38 MAPK in cancer development and therapy. Once activated, p38 proteins translocate from the cytosol to the nucleus, where they phosphorylate serine/threonine residues of many substrates, leading to the initiation of cellular stress responses or the promotion or inhibition of cancer cell growth, depending on the context. In some cases, p38 activation can lead to apoptosis (programmed cell death) and cell growth inhibition, whereas in others, it can promote cell proliferation and survival [8,41,42,43,44]. These findings suggest that tumor cell growth may be regulated by the coordination between cell proliferation and apoptosis.

Our experiments demonstrated that the glycosides significantly inhibited both total (p38) and phosphorylated p38 (p-p38) in MDA-MB-231 cells after 24 and 48 h of treatment, with a more pronounced effect at 48 h of treatment. The A_2B_AR antagonist PSB-1115 had no significant effect on p38 or p-p38 levels at 24 h but significantly reduced both at 48 h. In contrast, the receptor agonist NECA significantly suppressed total p38 levels at both time points. However, its effect on p-p38 was minimal, showing no significant change at 24 h and only 20% inhibition at 48 h compared to untreated cells.

The ERK pathway is a key intracellular signaling pathway involved in the regulation of cell growth, differentiation and apoptosis. Phosphorylated ERKs translocate to the cell nucleus, where they phosphorylate transcription factors, regulating the expression of genes required for cell growth, division, and survival. The reduction in ERK1/2 phosphorylation mediated by A_2B_AR might provide an interesting approach for adjuvant treatment, leading to reduced growth of certain tumors expressing the A_2B_AR, such as TNBC, where its overexpression is associated with worse prognosis.

The study of the influence of glycosides on the expression levels of ERK1/2 revealed that they significantly suppressed total and phosphorylated target proteins in MDA-MB-231 cells for both 24 and 48 h. PSB-1115 was more effective in inhibiting ERK1/2 at 48 h of exposure. NECA caused the strongest but opposite (stimulated) effect similarly after 48 h incubation.

Another member of the MAPK cascade, JNK1/2, was differentially affected by the glycosides, NECA, and PSB-1115. When the AR agonist significantly increased the levels of JNK1/2 and p-JNK1/2 at both time points (24 and 48 h), the antagonist slightly stimulated the expression of both JNK forms at the first time point, but then led to suppression of the total form but accumulation of the activated form. The influence of the glycosides also differed between them, especially after 48 h of exposure. Cuc A_0_-1 slightly increased both total and activated JNK1/2 at 24 h but strongly inhibited both forms at 48 h. Dj A did not affect JNK at either time point. The obtained results fit perfectly with the recently discovered mechanisms of the anticancer action of triterpene glycosides from sea stars, which specifically inhibit MAP kinases p38 and ERK1/2 [45]. Three independent methods have shown that the action of the AR agonist NECA led to an increase in the proliferation of MDA-MB-231 cells, which is consistent with the literature data [21]. These experiments also revealed that treatment with Cuc A_0_-1 led to a significant decrease in proliferation, whereas Dj A inhibited cell proliferation to a lesser extent. The selective A_2B_AR antagonist PSB-1115 did not directly affect cell proliferation. Co-treatment with glycosides and PSB-1115 significantly increased cell proliferation compared to glycoside treatment alone. This suggests that the antagonist blocks the adenosine receptor, potentially preventing glycosides from binding and exerting their antiproliferative effects. This is confirmed by the co-administration of NECA and PSB-1115, which preserved cell proliferation at the control level [21]. However, when glycosides were applied together with NECA, their antiproliferative effect increased. This may be explained by the non-selectivity of NECA in relation to the A_2B_ subtype AR. Glycosides bind most effectively to A_2B_AR that has been pre-activated by an agonist. We hypothesize that the subsequent increase in antiproliferative activity is due to biased signaling or “functional selectivity.” This concept suggests that different ligands can stabilize the same receptor in unique conformational states, leading to the activation of some signaling pathways while avoiding others [46]. The low affinity and non-specific nature of NECA for adenosine receptors is demonstrated by its relatively weak intermolecular interaction energy with A_2B_AR (−12.56 kcal/mol) (PDB ID 7XY7) [47]. Consequently, it can be readily displaced by higher-affinity, more specific ligands, such as PSB-1115, which has a significantly stronger interaction energy of −33.72 kcal/mol [48]. To support the experimental data on the anticancer action of triterpene glycosides via the A_2B_ adenosine receptor, molecular docking enhanced with short-term molecular dynamics simulations was implemented to model the binding pattern of glycosides with A_2B_AR. The in silico calculations were in good agreement with the cytotoxic activity of glycosides against MDA-MB-231 cells [19,30]. Hence, the highly cytotoxic compounds Cuc A_0_-1 and Dj A bound to both the orthosteric and allosteric sites of A_2B_AR by diverse modes, whereas the non-cytotoxic triterpene glycoside Dj D_1_ did not penetrate inside the receptor’s pocket and bound preferably to membrane lipids surrounding the A_2B_AR. 

Structure-based modeling revealed that six functionally significant residues (Met182, Met272, Val250, Val253, Phe173, and Lys267) of A_2B_AR are common for the binding sites of Cuc A_0_-1 and Dj A, despite the fact that the glycosides interact with them through different parts of their molecules. The TM6 residue Val253 and Lys267 on ECL3, which are unique to the A_2B_ARs subtype, are responsible for the selectivity of ligand recognition by ARs. The conservative residues Phe173 on ECL2, Met182 on TM5 helix, as well as and Met272 on TM7 formed VdW contacts with the aglycone of Cuc A_0_-1 or with the carbohydrate chain of Dj A, and are known to bind almost all both agonists and antagonists of A_2A_AR, for which the structures have been solved [33].

The results of molecular docking showed the extremely important role of VdW interactions for glycosides with the orthosteric binding site in the formation of complexes with A_2B_AR. Moreover, analysis of intermolecular non-covalent interactions revealed that Cuc A_0_-1 and Dj A formed energy-consuming HBs and/or hydrophobic interactions (contributing ≤ −1.0 kcal/mol) with the receptor residues Val250, Met272, Leu172, Phe173, which are thought to provide the high binding affinity and potency of selective A_2B_AR antagonist PSB-1115 [48,49]. Unlike Dj A, Cuc A_0_-1 showed interactions with Trp247, Ser279, Thr89, and water-mediated bridging HB with Asn254, which were predicted to be responsible for receptor binding to the antagonists PSB-1115 [48] and PSB-609 [50]. Interestingly, the importance of water molecules in the formation of bridging HBs with Asn254 and Phe173 residues for the A_2B_AR ligand to act as a potent antagonist has recently been demonstrated [51]. While terminal sugar residue of Dj A forms direct HB with Asn254 as well as a tight contact with Leu86 similar to PSB-1115. Cuc A_0_-1 and Dj A bind in distinct poses, forming unique interaction networks with the receptor. This results in a shallower binding pose and lower protein-ligand complementarity for Dj A (Table S1, Figure S1), explaining its higher estimated binding energy (–28.64 kcal/mol vs. –33.77 kcal/mol for Cuc A_0_-1). This structural insight was experimentally confirmed by the greater antiproliferative potency of Cuc A_0_-1.

In silico modeling of Dj D_1_ binding with A_2B_AR showed that its carbohydrate chain preferentially formed strong bonds with phospholipid heads of membrane lipids and apparently facilitated the aglycone incorporation into the inner part of the bilayer, which was confirmed earlier by the ability of the glycoside to destroy the erythrocyte membrane but not affect cancer cells [30,52]. Hence, despite an extensive network of non-covalent interactions (ionic, HBs, hydrophobic, VdW) (contributing up to –14.86 kcal/mol) and the participation of TM3 receptor helix (H-π −0.4 kcal/mol and VdW –3.75 kcal/mol) and ECL2 loop (−11.76 kcal/mol, HBs, hydrophobic and VdW) in the glycoside binding, none of interactions with functionally important residues responsible for recognizing of agonists or antagonists by ARs were found for Dj D_1_.

We recently applied a ligand-based approach to define the structure-activity relationships (SAR) governing the cytotoxicity (pIC_50_) of glycosides against MDA-MB-231 cells [20]. The results from the present structure-based study align excellently with this prior QSAR model.

The model identified several features that significantly enhance cytotoxicity: an 18(20)-lactone, a normal side chain, a 16-O-acetyl group, and a 23-ketone moiety in the aglycone, as well as a sulfate group at C-4 of the first xylose residue. Crucially, our docking simulations show that all these groups are directly involved in Cuc A0-1’s interaction with A_2B_AR and make a high contribution to its favorable binding energy.

Conversely, branching in the carbohydrate chain (e.g., in Cuc A_0_-1 and Dj D_1_) negatively correlates with cytotoxicity compared to linear chains (e.g., Dj A). Although the terminal (branching) xylose unit in Cuc A_0_-1 forms two hydrogen bonds with Glu6, a significant steric obstacle results in a net positive energy contribution (+3.46 kcal/mol), indicating that this branch impedes binding. This suggests that the branched chain may force Cuc A_0_-1 to bind primarily via its aglycone, whereas the linear chain of Dj A allows it to enter the binding site. The free hydroxyl group at C-2 of the quinovose residue in Dj A participates directly in A_2B_AR binding. Additionally, the methyl group of quinovose helps maintain a conformation that favors electrostatic attraction to the allosteric site. Similarly, the 3-*O*-methyl group of glucose was incorporated directly into the binding site, explaining its positive effect.

Furthermore, previous SAR analyses have indicated that an increase in hydroxyl groups, particularly in the aglycone and at specific carbohydrate chain positions, decreases activity [20]. This is confirmed by Dj D_1_ binding, where the substitution of quinovose with glucose and chain branching drastically reduced its affinity for A_2B_AR. Finally, MD simulations refined the docking results, revealing that hydroxyl groups at C-2 and C-4 of the xylose residue and at C-4 and C-6 of 3-*O*-methylglucose in Dj A interact directly with both conserved residues (important for general AR modulation) and variable residues specific for A_2B_AR ligand recognition.

**Figure 10:**
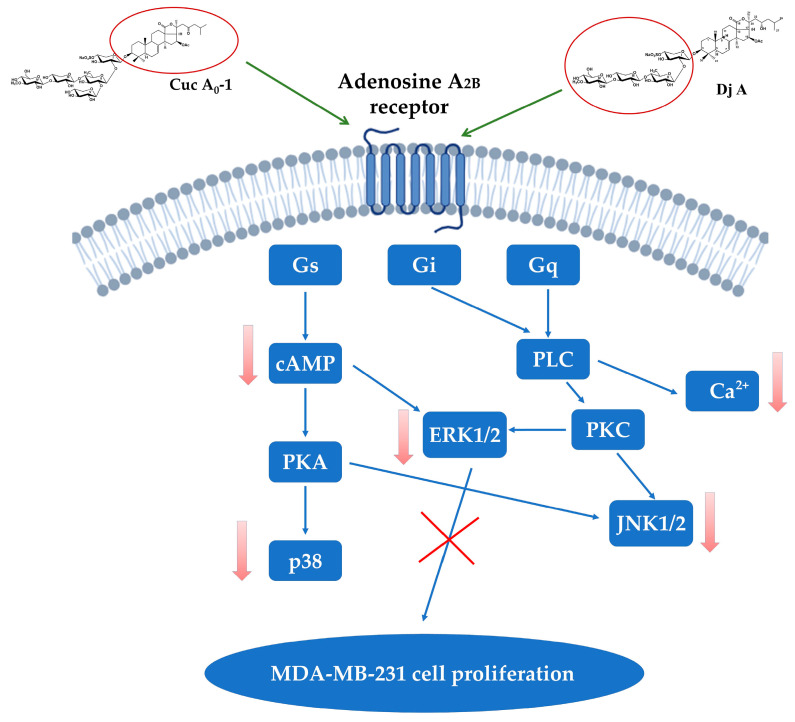
Adenosine A2B receptor signaling triggered by triterpene glycosides in MDA-MB-231 cells.

It has been established that triterpene glycosides act as biased antagonists of the A_2B_ adenosine receptor triggering MAPK signaling. The interaction of glycosides with the NECA-preactivated receptor may bias signaling toward the Gi and Gq/PLC/ERK1/2 pathways, potentially leading to antiproliferative or apoptotic effects. The potent suppression of ERK1/2 by the tested glycosides strongly correlated with their significant antiproliferative effect, underscoring the central role of the MAPK pathway in controlling cell growth (Figure 10). This explains their unique effects, including the activation of the intrinsic pathway of apoptosis, increase in ROS levels, and change in Bax/Bcl-2 levels. These findings are of considerable fundamental importance, revealing a new ligand class for a therapeutically relevant receptor and establishing novel structure-activity relationships (QSAR), while also demonstrating direct practical applicability. Identifying the adenosine receptor as the target of triterpene glycosides in TNBC MDA-MB-231 cells underscores the need for further investigation to fully unravel their mechanism of action. Future studies will therefore comprehensively profile these glycosides across a panel of TNBC cell lines (e.g., MDA-MB-468, BT-549, SUM149PT, HCC1187) and evaluate their in vivo efficacy, both as monotherapy and in combination with standard chemotherapeutics. Further plans include a detailed analysis of the binding affinity, kinetics, mechanism, and selectivity of the glycosides for the A_2B_AR utilizing surface plasmon resonance (SPR).

## 4. Materials and Methods

### 4.1. Reagents and Antibodies

Cucumarioside A_0_-1 (Cuc A_0_-1) and Djakonovioside A (Dj A) were isolated from the sea cucumber *Cucumaria djakonovi*, and their purity was confirmed by ^1^H and ^13^C NMR spectroscopy and HR-ESI mass spectrometry [19]. The compounds were dissolved in ddH_2_O at a concentration of 1 mM and stored at +4 °C until use.

MEM medium (Biolot, St. Petersburg, Russia)*,* fetal bovine serum (Biolot, St. Petersburg, Russia)*,* penicilin-streptomycin (Biolot, St. Petersburg, Russia)*,* RIPA buffer (Sigma-Aldrich, St. Louis, MO, USA), PSB-1115 (Macklin, Shanghai, China), NECA (Calbiochem, San Diego, CA, USA), ELISA kit for cAMP (CEA003Ge, Cloud-Clone, Wuhan, Hubei**,** China), BSA (Sigma-Aldrich, St. Louis, MO, USA), ECL solution (Bio-Rad, Hercules, CA, USA), FluoriCa-8 AM (Lumiprobe, Moscow, Russia) were used. Primary antibodies (Affinity, Shanghai, China), at a titer of 1:1000, were used in the experiments: monoclonal mouse antibodies against p38 MAPK; polyclonal rabbit antibodies against phospho-p38 MAPK; polyclonal rabbit antibodies against ERK1/2; polyclonal rabbit antibodies against phospho-ERK1/2; polyclonal rabbit antibodies against JNK1/2/3; and polyclonal rabbit antibodies against phospho-JNK1/2/3. Monoclonal mouse antibodies against beta-actin (titer 1:1000), secondary goat antibodies labeled with horseradish peroxidase against rabbit, titer 1:10,000 and secondary goat antibodies labeled with horseradish peroxidase against mouse, titer 1:10,000 (Cloud-Clone, Wuhan, Hubei**,** China) were used.

### 4.2. Cell Lines and Culture Conditions

The human triple negative breast adenocarcinoma MDA-MB-231 cell line was obtained from ATCC (HTB-26**^™^**, Manassas, VA, USA). MDA-MB-231 cells were cultured as a monolayer under standard conditions (37 °C, 5% CO_2_) in MEM medium supplemented with 10% fetal bovine serum and 1% penicillin-streptomycin.

### 4.3. ELISA

MDA-MB-231 cells were seeded in 6-well plates (5 × 10^4^/^mL^) and incubated for 24 h (37 °C, 5% CO_2_) until complete adhesion. Then 1 μM of Cuc A_0_-1, 2 μM of Dj A, and 1 μM of PSB-1115 were added to the cells for 6 h of incubation. Cells incubated without compounds were used as a control. Then, Ripa-buffer was added to the cells for lysis (10,000× *g*, 15 min, 4 °C). The supernatant from the cell lysates was collected and immediately analyzed using a cAMP kit (CEA003Ge, Cloud-Clone, Wuhan, Hubei, China), according to the manufacturer’s instructions.

### 4.4. Calcium Uptake

MDA-MB-231 cells were plated in 96-well plates at a density of 10 × 10^3^ cells per well in MEM medium and incubated overnight at 37 °C in a 5% CO_2_ atmosphere. The cells were then washed once with HBSS (pH 7.4) and subsequently loaded with 5 μM FluoriCa-8 AM (Lumiprobe, Moscow, Russia) in the same buffer. After loading, the cells were incubated for 40 min at 37 °C in a 5% CO_2_ environment, washed with HBSS saline without the fluorescent dye, and treated with Cuc A_0_-1 (1 μM), Dj A (2 μM), and PSB-1115 (1 μM) for 30 min at 37 °C in a 5% CO_2_. The standard agonist NECA (final concentration 1 μM) was added via a robotic microinjector following baseline recording. in each well for Ca^2+^ influx stimulation. Fluorescence intensity was recorded using a PHERAstar FS plate reader (BMG LABTECH, Ortenberg, Germany) by measuring the excitation at 490 nm and emission at 510 nm. Data were analyzed using MARS Data Analysis version 3.01R2 (BMG Labtech, Ortenberg, Germany).

### 4.5. Western Blotting

MDA-MB-231 cells (1 × 10^6^ cells/well) were seeded into Petri dishes and incubated for 24 h to allow adhesion. Then, 1 μM Cuc A_0_-1, 2 μ Dj A, 1 μM NECA, or 1 μM PSB-1115 were added and incubated with the cells for an additional 24 and 48 h. The cells were then collected and lysed using RIPA buffer. The protein lysates were subjected to electrophoresis in Precast gel Plus Tris-Gly 4–15% gel (WSHT, Shaanxi, China) and transferred to a PVDF membrane (0.45 μm, Millipore Corporation, Burlington, MA, USA). Afterwards, the membrane was blocked in a solution of 5% BSA for 1 h and then incubated with primary antibodies overnight at 4 °C, and incubated with secondary antibodies for 1 h at room temperature. Detection was performed using an ECL solution and a ChemiDoc MP imaging system (Bio-Rad, Hercules, CA, USA).

### 4.6. Antiproliferative Activity

MDA-MB-231 cells were plated in 12-well plates at a density of 100 cells per well in MEM medium and incubated overnight at 37 °C in a 5% CO_2_ atmosphere. Then, substances were added to the cells: 1 μM of Cuc A_0_-1; 2 μM of Dj A; 1 μM of PSB-1115 and 1 μM of NECA. Cells were incubated for 9 days in a CO_2-_incubator at 37 °C, detached from the plate, and counted under an inverted microscope (AxioVert, Carl Zeiss, Göttingen, Germany).

To determine the antiproliferative activity using the MTS method, MDA-MB-231 cells were seeded in 96-well plates at 50 cells per well. The glycosides, NECA and PSB-1115, were added 24 h later. After 9 days, 10 μL of the MTS reagent was added to each well of the 96-well plate, incubated for 4 h, and the absorbance in each well was determined at 490/630 nm using a PHERAstar FS plate reader (BMG Labtech, Ortenberg, Germany).

For colony formation testing, MDA-MB-231 cells were plated in 6-well plates (150 cells/well) and incubated overnight to allow attachment. After that, solutions of the study compounds were added to the cells: Cuc A_0_-1—1 μM, Dj A—2 μM, PSB-1115—1 μM, and NECA—1 μM, either alone or in combinations and incubated for 14 days in a CO_2_ incubator. The colonies were fixed with methanol, stained with Giemsa reagent, and counted using a BIO-PRINT-Cx4 Edge-Fixed Pad-Container (Vilber, Collegien, France) and Bio-Vision Software 2.1 user and service manual-v18.01 (Vilber, Collegien, France). For the analysis of areas of overgrowth of the well with colonies, the free image processing version Fiji (ImageJ, 1.53t, Wayne Rasband and contributors National Institutes of Health, USA) was used. The results are presented as mean values as a percentage of the control.

### 4.7. Modeling of A_2B_AR in Lipid Environment and Molecular Docking of Glycosides

The human A2BAR model was constructed, including extracellular and intracellular loops ECL2, ECL3 and ICL3, correspondingly. The coordinates of the A_2B_AR atoms were extracted from the cryogenic electron microscopy (cryo-EM) structure of the receptor coupled to a modified Gs protein resolved at 3.2 Å (PDB ID 8HDP [32]. Extracellular loop modeling was performed using the Loop Modeling Tool of the MOE 2019.01 CCG package [27]. The protein model was inserted into an asymmetric mammalian plasma membrane (lipid membrane composition is presented in Table S2) using the insertion method on the CHARMM-GUI web server (available online: http://www.charmm-gui.org (accessed on 18 June 2025) [28,29]. Each leaflet contained approximately 400 lipids. The outer leaflet composition consisted of phosphatidylcholine (POPC, PLPC), phosphatidylethanolamine (POPE, PAPE), phosphatidylserine (POPS), sphingomyelin (SSM, NSM), and cholesterol (CHOL), whereas the inner leaflet contained the same lipids, with phosphatidylinositol (POPI), phosphatidic acid (POPA), and phosphatidylserine (PAPS) [53].

To predict the most probable glycosides binding modes and conformations, a blind molecular docking procedure was performed using the protein–ligand docking protocol with London dG Scoring function (30,000 poses were generated) followed by Post-Docking Refinement using Affinity dG Scoring with MOE 2019.01 CCG software [27]. The A_2B_AR model inserted into an asymmetric mammalian plasma membrane was used as the docking receptor. Cuc A_0_-1, Dj A, or Dj D_1_ molecules were placed according to the most energetically favorable positions obtained from the molecular docking results for each. The starting system was protonated in an Amber 14:EHT force field at pH 7.0 using MOE 2019.01 CCG with the semiempirical molecular orbital MOPAC 7 suite for glycoside partial charge calculations, solvated with approximately 43,600 water molecules in 1 M KCl (48–64 K^+^ and 6–10 Cl^−^ depending on the system). All-atomic MD simulations of 20 ns-long were carried out at constant pressure (1 atm) and temperature (300 K) with a time step of 2 fs. The all-atomic force field Amber 14:EHT was used for proteins [54] and lipids [55]; for water, and TIP3P was used [56]. The solvent molecules were treated as rigid molecules. MD simulations, including heating, equilibration, and energy minimization steps, were performed using the MOE2019.01 CCG software package [27]. The contributions of noncovalent intermolecular interactions to the free energy of complex formation were also estimated.

### 4.8. Statistical Analysis

All experiments were performed in triplicates. Data were subjected to statistical analysis using one-way analysis of variance (ANOVA) tests. Data are shown as mean ± SEM, and *p* ≤ 0.05 was regarded as statistically significant. All statistical tests were performed using SigmaPlot 14.0 software (Systat Software Inc., San Jose, CA, USA).

## 5. Conclusions

Triterpene glycosides Cuc A_0_-1 and Dj A were identified as novel antagonists of the adenosine A_2B_ receptor (A_2B_AR), which is overexpressed in triple-negative breast cancer MDA-MB-231 cells. This mode of action was functionally confirmed by the ability of the glycosides to reduce intracellular cAMP levels and block Ca^2+^ influx induced by the canonical A_2B_AR agonist NECA. Molecular modeling revealed that both glycosides bind to A_2B_AR but engage in distinct interaction modes: Cuc A_0_-1 primarily utilizes its aglycone moiety, whereas Dj A employs its carbohydrate chain. These interactions span both orthosteric and allosteric sites, effectively modulating the functional state of the receptor. Consequently, glycoside binding inhibits downstream MAPK signaling pathways, ultimately leading to the suppression of TNBC cell growth and the induction of apoptosis. To our knowledge, this is the first study to establish A_2B_AR as a direct target of sea cucumber-derived triterpene glycosides in MDA-MB-231 cells. Their potent antitumor effects, mediated through the antagonism of A_2B_AR and subsequent MAPK pathway inhibition, position them as promising lead compounds for cancer types with high expression A_2B_AR.

## Data Availability

The original data are available from the corresponding author upon request.

## Supplementary Materials

The following supporting information can be downloaded at: https://www.mdpi.com/article/10.3390/ijms262110327/s1.
