# Supplementary material for: Anticancer Activity of Triterpene Glycosides Cucumarioside A0-1 and Djakonovioside A Against MDA-MB-231 as A2B Adenosine Receptor Antagonists
Source: Int J Mol Sci. 2025 Oct 23;26(21):10327. doi: 10.3390/ijms262110327 (PMC12607671; doi:10.3390/ijms262110327)
Supplement: Supplementary file 1 [file ijms-26-10327-s001.zip › ijms-3890424-supplementary.pdf]

Supplementary Data

Title: Anticancer activity of triterpene glycosides Cucumarioside A_0-1 and Djakonovioside A against human TNBC as A2b adenosine receptor antagonists inhibiting the MAP-Kinase pathway.

Content:

Table S1. Total SASA loss under complex formation.

Figure S1. Structural diagram of Cuc A_0-1 **(a)**, Dj A **(b)**, Dj D_1 **(c)** binding with A2bAR in lipid environment.

Figure S2. The backbone root mean square fluctuation (RMSF) plots for A2bAR in lipid environment under binding with glycosides.

Table S2. Asymmetric mammalian plasma membrane lipid membrane composition.

Figure S3. Images of original uncropped Western blots: ERK1/2 and p-ERK1/2; β-actin; p38 and p-p38; JNK1/2 and p-JNK1/2 for 24 h.

Figure S4. Images of original uncropped Western blots: ERK1/2 and p-ERK1/2; β-actin; p38 and p-p38; JNK1/2 and p-JNK1/2 for 48 h.

Table S1. Total SASA loss under complex formation.

Ligand	Docking pose			20nc MD simulation		
	$\Delta SASA_L$, Å^2	$\Delta SASA_R$, Å^2	S_{compl}	$\Delta SASA_L$, Å^2	$\Delta SASA_R$, Å^2	S_{compl}
Cuc A_0-1	669.6	804.4	0.64	746.7	869.4	0.71
Dj A	558.6	672.9	0.56	605.8	698.3	0.68
DjD1	530,2	619,6	0,39	264,3	671,8	0,42

$\Delta SASA_L$– the ligand SASA loss in complex formation, $\Delta SASA_R$– the receptor SASA loss in complex formation, $A_{2B}AR$ in the membrane environment is considered as a receptor. S_{compl} –The total shape complementarity at the interface.

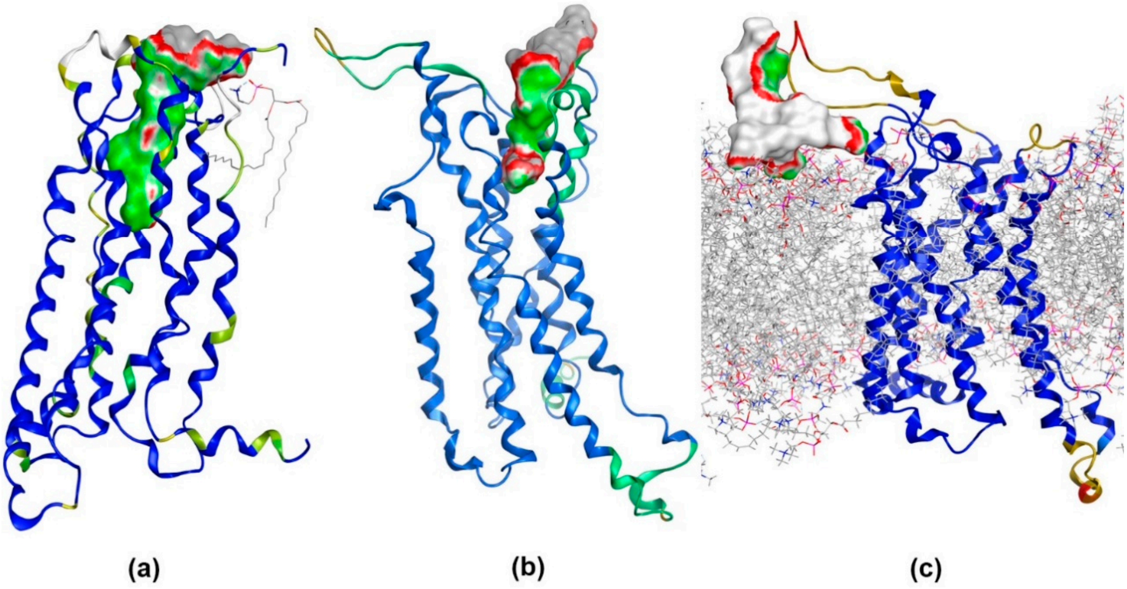

Figure S1. Structural diagram of Cuc A_0-1 **(a)**, Dj A **(b)**, Dj D_1 **(c)** binding with A2bAR in lipid environment. A2bAR is represented as ribbon and colored according to RMSF value (from blue (1Å) to red (4 Å)), an asymmetric mammalian plasma membrane lipid – as gray sticks, glycosides are presented as molecular surface and colored according to surface complementarity (S_{compl}): green – high S_{compl} (0.9), mid S_{compl} (0.4) – white, low S_{compl} (0.1) – red, and solvent exposed – gray. The aqueous environment and some lipid membrane components were removed for clarity.

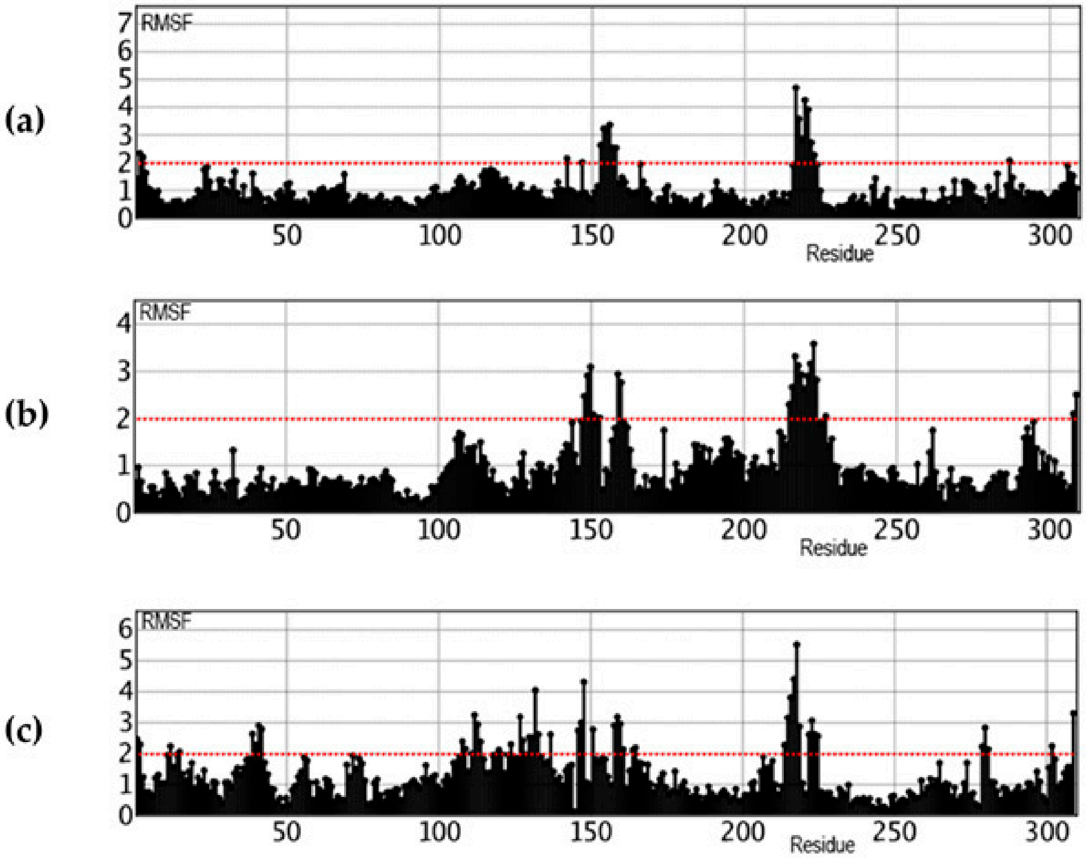

Figure S2. The backbone root mean square fluctuation (RMSF) plots for A2bAR in lipid environment under binding with **(a)** Cuc A_0-1, **(b)** Dj A, **(c)** Dj D_1.

Table S2. Asymmetric mammalian plasma membrane lipid membrane composition.

Lipid Name	Lipid Head/Tail	Outer Leaflet	Inner Leaflet
POPC	PC(16:0/18:1(9Z))	64	28
PLPC	PC(16:0/18:2(9Z,12Z))	88	44
PAPE	PE(16:0/20:4(5Z,8Z,11Z,14Z))	12	48
POPE	PE(16:0/18:1(9Z))	12	56
POPI	PI(16:0/18:1(9Z))	0	20
PAPS	PS(16:0/20:4(5Z,8Z,11Z,14Z))	0	44
POPA	PA(16:0/18:1(9Z))	0	4
SSM	SM(d18:1/18:0)	44	20
NSM	SM(d18:1/24:1)	44	20
CMH	GlcCer(d18:1/16:0)	16	0
CHOL	Cholesterol	148	116
TOTAL		428	400

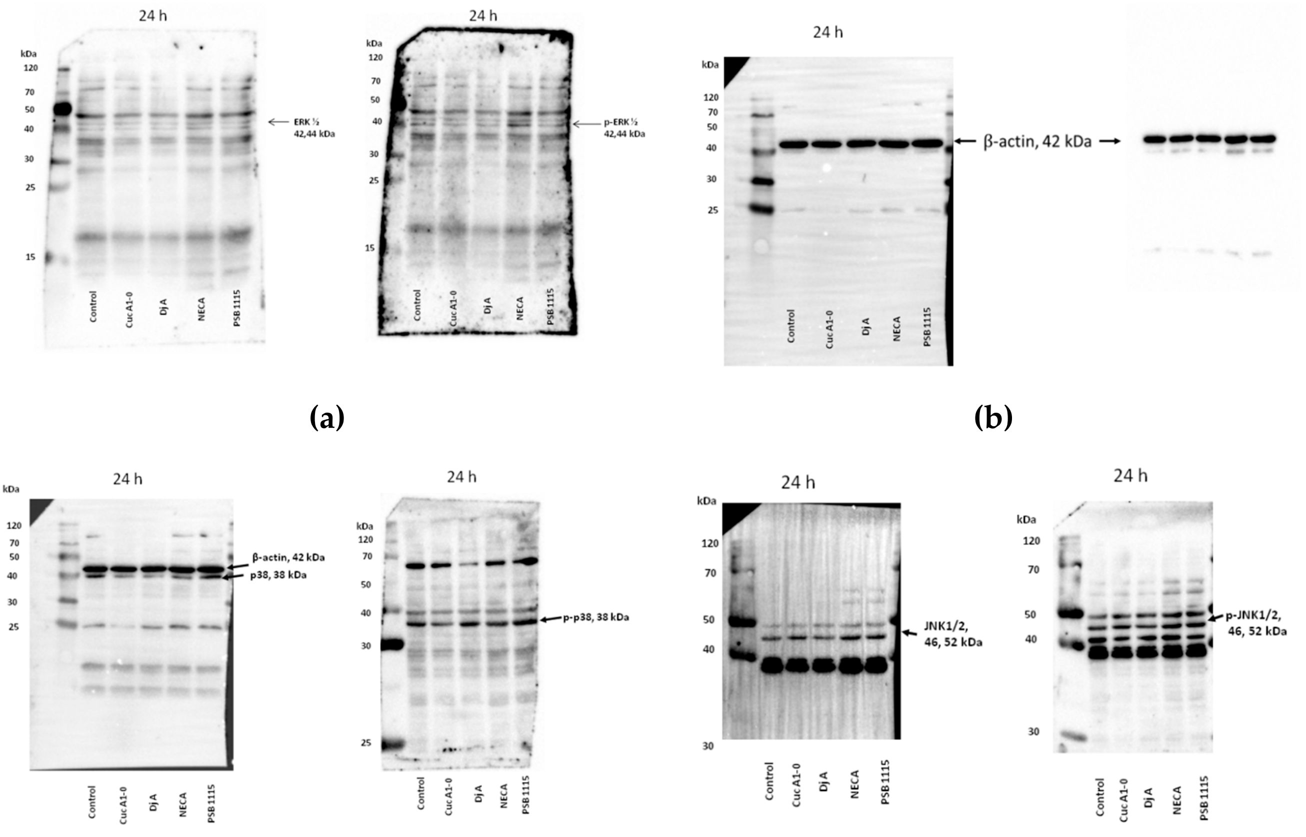

Figure S3. Images of original uncropped Western blots displayed in this manuscript**: (a)** ERK1/2 and p-ERK1/2; **(b)** β-actin; **(c)** p38 and p-p38; **(d)** JNK1/2 and p-JNK1/2. Protein extracts were obtained from MDA-MB-231 cells incubated with the substances for 24 h.

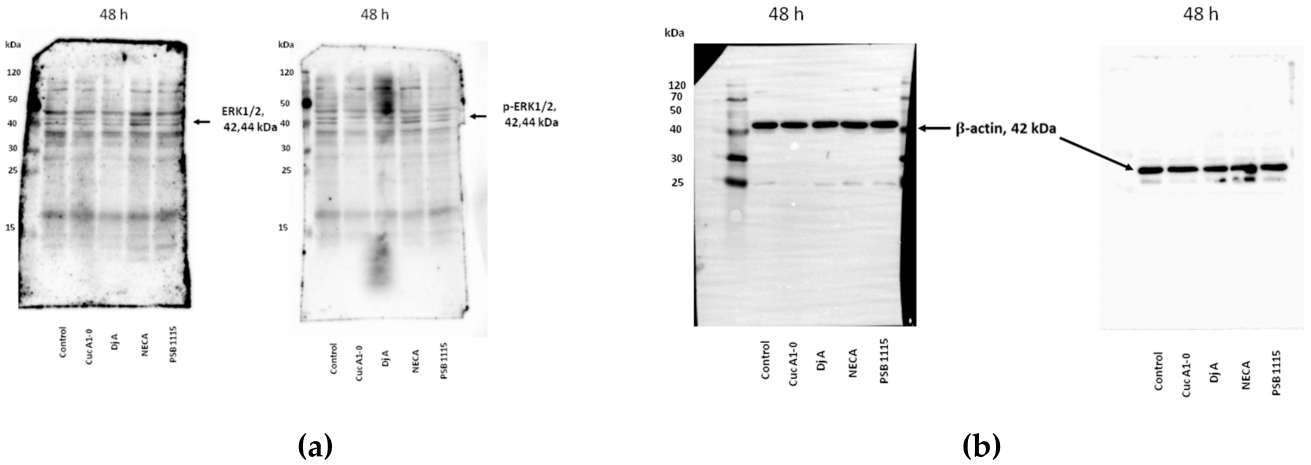

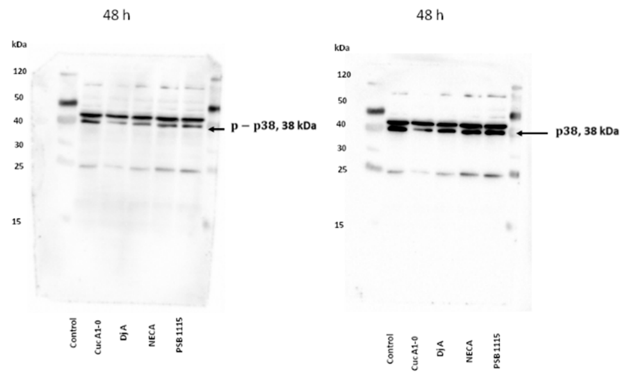

(c)

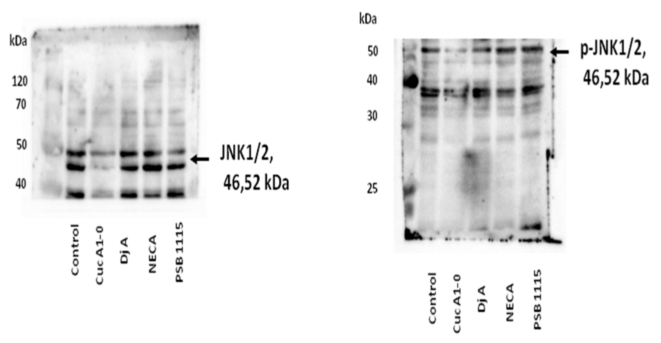

(d)

Figure S4. Images of original uncropped Western blots displayed in this manuscript: **(a)** ERK1/2 and p-ERK1/2; **(b)** b-actin; **(c)** p38 and p-p38; **(d)** JNK1/2 and p-JNK1/2. Protein extracts were obtained from MDA-MB-231 cells incubated with the substances for 48 h.
